# Orbital blood vessels changes on color duplex imaging in diabetics with and without diabetic retinopathy

**DOI:** 10.1038/s41598-023-43838-9

**Published:** 2023-10-10

**Authors:** Alia M. Noureldine, Aya Montasser Sayed Abdelmaksoud, Hisham Adel Abdel Fatah Mostafa, Tamer Macky, Abo Elmagd ElBohy

**Affiliations:** 1 Ophthalmology Department, Kasr Alainy School of Medicine, Cairo University, Cairo, 11956 Egypt; 2 Diagnostic and Interventional Radiology, Kasr Alainy School of Medicine, Cairo University, Cairo, Egypt

**Keywords:** Eye diseases, Metabolic disorders

## Abstract

To compare changes in ophthalmic artery (OA) and its branches in diabetics with and without diabetic retinopathy (DR) using color duplex imaging (CDI), and to correlate these changes with the disease variables. 60 eyes of 60 diabetic patients were enrolled, divided into 3 groups: without DR (Group A), with Non-Proliferative DR (Group B) and with Proliferative DR (PDR) (Group C). Laboratory testing including HbA1c was done. Patients underwent CDI, by which OA, Central Retinal Artery (CRA) and Ciliary Arteries were identified; for each of them we measured Peak systolic velocity (PSV), End Diastolic velocity (EDV) and Resistivity Index (RI). Results were compared to clinical, laboratory and fundus examination. OA EDV was significantly lower and OA RI was found to be significantly higher in Group C (*p* = 0.027 and 0.025 respectively). CRA PSV and EDV were significantly lower in Group C (*p* = 0.017 and 0.001 respectively). PCA RI was significantly higher in Group C (*p* = 0.008). HbA1c was negatively correlated with CRA PSV (*p* = 0.041), also it was negatively correlated with CRA EDV (*p* = 0.0001), as well as with PCA EDV (*p* = 0.002). There was direct significant correlation between HbA1c and PCA RI (*p* = 0.012). Duration since diagnosis was negatively correlated with CRA EDV (*p* = 0.004). Multivariate linear regression showed that DR is an independent predictor for low OA EDV, high OA RI, low CRA EDV and high PCA RI. DR is an independent risk factor for orbital and ocular vessels flow alteration, thus can be used as a prognostic tool in diabetic patients. CDI can be reliably used in diabetics to predict early changes or progression of DR.

## Introduction

Till now, the actual pathogenesis behind the Diabetic Retinopathy (DR) onset and progression remains somehow unrevealed despite years and years of research. Hemodynamically, evidence has concluded that there is an early decrease in retinal blood supply even before the onset of DR^1^. Studying hemodynamics of retrobulbar vessels in diabetes using CDI can help in understanding the pathogenesis and can give an insight into the prospective treatment for DR. Moreover, it can guide us to predict individuals at advanced risk of developing vision threatening complications. Several studies have used CDI to analyze the circulatory changes in diabetics and found contradictory results: while some have noted a decrease in the retrobulbar blood flow velocities, others have reported an increase or no change in the same parameters^1^.

**Figure 1:**
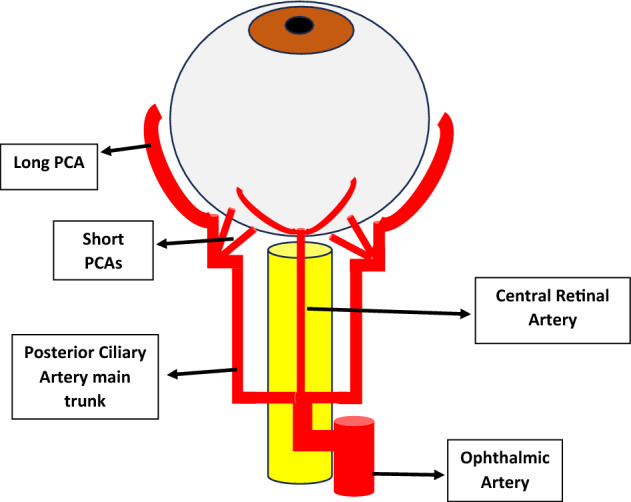
Hand drawn diagram showing the ocular blood supply and vessels analyzed by CDI.

Blood supply of the retina comes from two branches of the Ophthalmic Artery (OA): the central retinal artery (CRA) and the posterior ciliary arteries (PCA)^2^. The CRA, running along the optic nerve, pierces the lamina cribrosa and enters the optic disc nasal to the posterior pole. It then divides into superior and inferior branches, which continue to bifurcate to supply blood to the inner retinal layers^2^. The CRA is an end branch of the OA and is considered as the main blood supplier to the retina. As for the outer retina, it is nourished through the PCA, which arises as a main trunk from OA then divides into short PCA and 2 long PCA that run anteriorly to supply CB and iris (Fig. 1).

The retro-ocular blood vessels that can be assessed by CDI include the OA, the PCA, CRA & CRV. CDI can also be used to study intraocular blood vessels, including the vortex veins, ocular tumors vasculature, vitreoretinal neovascular membranes as well as vessels in detached retina^3^. Due to its ability to analyze these vessels qualitatively and quantitatively, numerous studies have assessed changes in ocular blood flow in different eye diseases such as DR, glaucoma, CRA & CRV occlusions and have revealed significant differences compared to healthy individuals even in early disease stages^4^. In this study, we measured the different CDI parameters of the orbital vessels in diabetics with and without retinopathy in an attempt to detect correlations with different disease variables. DM affects roughly 15.6% of Egyptians between the ages of 20 and 60 years old^5^. DR affects 17.9–20% of diabetic individuals, according to some estimates^6,7^. CDI may be ideal for longitudinal follow up of diabetic patients for early detection of hemodynamic changes caused by ischemic retinopathy and hence early timely intervention to abort its progression. Some studies even estimated that CDI may replace fundus examination as the latter is relatively time consuming and annnoying to the patient because it needs prior dilatation of the pupil and causes blurry vision for several hours^8^. We do not agree with this opinion, despite our agreement that CDI may be of great use in early prediction of diabetic vasculopathy progression.

## Patients and methods

This is a cross sectional study on diabetic patients at the ophthalmology outpatient clinic of Kasr Alainy hospital, Cairo University, between November 2020 to July 2021. All patients received a thorough explanation of the study design and aims. An informed consent was obtained from all patients. The study protocol was revised and approved by the Research Ethics Committee, Faculty of Medicine, Cairo University (Code:MS-454-2020) and followed the tenets of the Helsinki declaration.

### Study population

#### Inclusion criteria

Diabetic patients with more than 5-years duration were included. Only 1 eye per subject was selected in the study, the eye with more diabetic changes was chosen in case of patients with retinopathy, while in diabetics with no fundus changes, it was randomly selected.

#### Exclusion criteria

We excluded patients with vascular retinopathies other than DR (e.g., hypertensive retinopathy, retinal artery or vein occlusion, hereditary systemic angiopathy); patients with glaucoma, laser photocoagulation, previous intravitreal injections, recent eye trauma or surgery, congenital anomalies, any inflammatory process involving the eye and globe. Patients suspected to have hypoglycemic coma or diabetic keto acidosis, patients with any other hormonal disturbance rather than DM, cardiovascular patients and chronically ill (e.g., nephropathy) patients were also excluded. Also, patients who take any medications affecting the circulation hemodynamics such as lowering blood lipids and preventing platelet aggregation, were excluded.

### Data collection

Patients had their demographics (age and gender) recorded, and a review of their medical history including personal history, history and duration of systemic diseases, history of ocular diseases. All patients underwent random blood sugar and glycosylated hemoglobin level (HbA1c) testing and the duration of diabetes mellitus (DM) was noted. All patients had comprehensive ophthalmological examination, including Best Corrected Visual Acuity (BCVA) using Snellen’s chart, slit lamp examination, assessment of intraocular pressure by Goldmann’s applanation tonometry, and dilated fundus examination by binocular indirect slit-lamp biomicroscopy. In patients with retinopathy, Fundus Fluorescein Angiography (FFA) was done to detect possible proliferation if not seen clinically. Patients were divided into 3 groups. Group A: without diabetic retinopathy, Group B: with Non-Proliferative Diabetic Retinopathy (NPDR), and Group C: with PDR. All selected patients had no previous treatment.

### Color duplex imaging

CDI scan was performed to all patients using the same machine by a single experienced operator. The operator was blind to the clinical and fundus examination findings of the patients. The study was performed using the ultrasound and Color duplex machine (TOSHIBA TUS A-500, Toshiba medical systems corporation, JAPAN) using linear array probe (L11-13 MHZ). Patients were placed in supine decubitus, instructed to keep their eyes straight ahead while closing their lids. A good amount of acoustic coupling gel was applied to the closed upper eyelid and the probe was gently positioned, avoiding exerting excessive pressure on the eye and the hand of the operator rested either on the patient’s forehead or cheek for fine manipulation of the probe. We used the preset for small parts which allowed detection of low flow with low pulse repetition frequency, low filter and high gain. First, we identified the eye globe using the *B- mode* image, then the optic nerve was identified at the posterior aspect of the eye globe as the hypo-echoic structure, and used as a landmark and depth was adjusted to visualize the apex of the orbit then the overall gain, time gain compensation and focus were adjusted to visualize the whole orbital cavity and its contents clearly. Next, *Color mode* was applied, adjusting velocity scale to 7–15 cm/second using low filter setting and increasing color gain till noise appears then slightly decreasing the gain to just below the noise floor.

**Figure 2:**
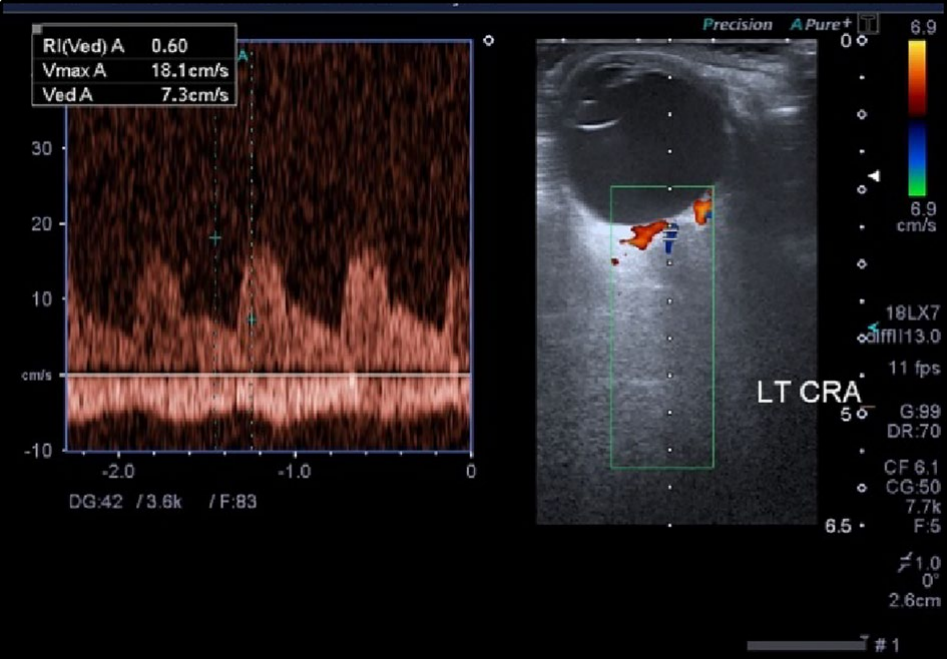
CDI of the left eye of a diabetic patient showing the Doppler spectral waveform of CRA and CRV. The flow velocity waveform in the CRA is of low amplitude and typical peaks for systolic and diastolic velocities are seen above the zero line. The flow velocity waveform in the CRV (almost monophasic) presents below the zero line.

**Figure 3:**
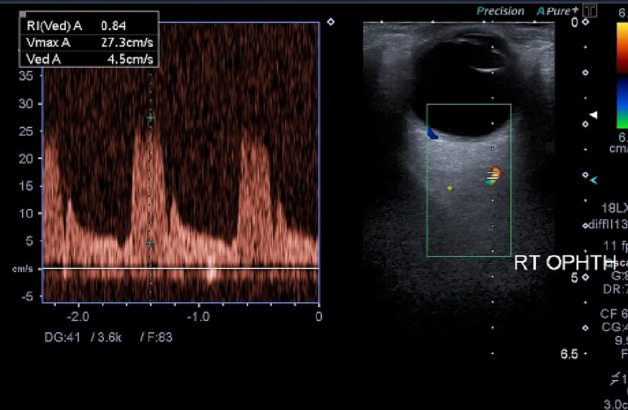
CDI of the right eye of a diabetic patient showing the Doppler spectral waveform of OA at the intersection of the OA with the Optic Nerve. Note the characteristic waveform of the OA showing the high-resistance flow with high systole and low diastole.

Central Retinal Artery (CRA) was identified running within the optic nerve together with the Central Retinal Vein (CRV) (Fig. 2), while Ciliary Arteries (CA) (Nasal or Temporal were identified on either both sides of the optic nerve and Ophthalmic Artery (OA) was identified arising at the apex of the orbit temporally and crossing nasally over (or below) the optic nerve (Fig. 3). Regarding CA, they arise as nasal and temporal trunks from the OA, then they divide into short and long branches. We scanned PCA at the main trunk before their division. Since the angle can't be fixed at zero in advance, the cursor was kept parallel or near parallel the direction of blood flow (according to the vessel direction), usually at an angle minor than 60°. For each artery, we measured Peak Systolic Velocity (PSV), End Diastolic Velocity (EDV), and Resistivity Index (RI) while adjusting sample volume to 1 mm, angle at zero and CDI filter at 70 Hz (low filter setting) and again the gain was increased until the noise appeared in the CDI spectrum then decreased again just below the noise floor.

### Outcome measures

1. Comparing CDI parameters (PSV, EDV and RI) of OA, CRA and PCA between Groups A, B and C.
2. Correlation between measured CDI parameters (PSV, EDV and RI) and disease variables including age, duration of DM, HbA1c level, and presence of retinopathy.
3. Multivariate Linear Regression Analysis was performed with different dependent variables (OA EDV, OA RI, CRA PSV, CRA EDV and PCA RI) and independent variables such as age, sex, duration of diabetes and DR.

### Statistical analysis

Statistical analysis was conducted using SPSS 22nd edition, numeric variables were presented in mean ± standard deviation and were compared using Mann Whitney u test between 2 groups and Kruskal Wallis test between > 2 groups after normality testing. Categorical variables were presented in frequency and percentage and were compared using Chi2 test. Spearman correlation test was used to assess the correlation between 2 numeric variables. Any *p* value < 0.05 was considered significant.

## Results

**Figure 4:**
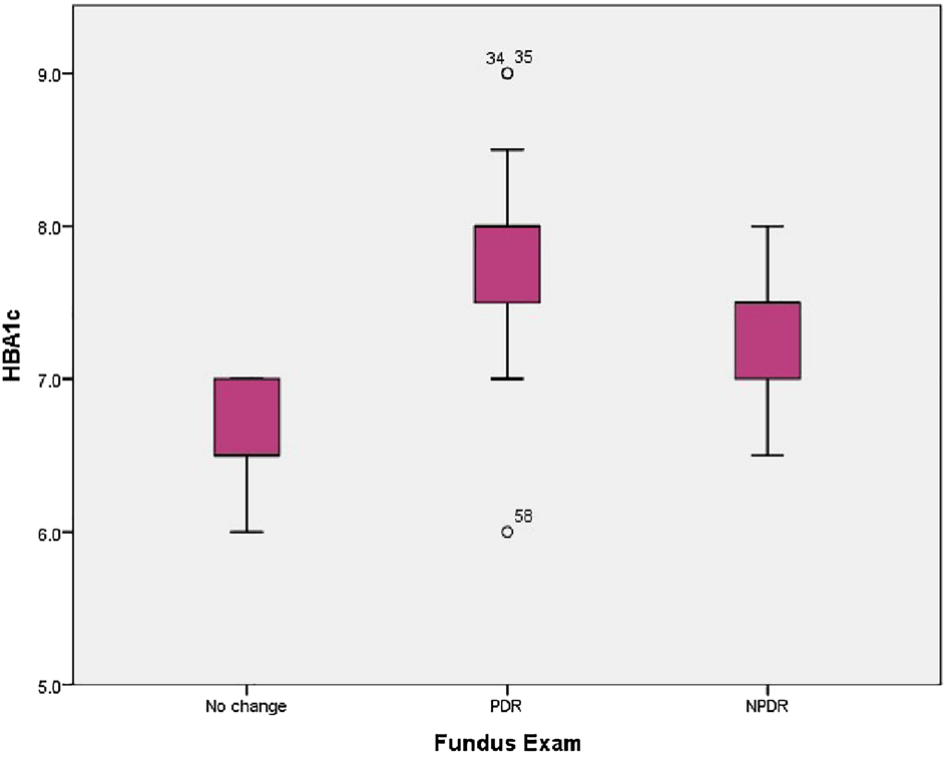
Box plot showing HbA1c distribution between the 3 groups. No change = Group A, Group B = NPDR, Group C = PDR.

We have included 60 diabetic patients in our study with a mean age of 53 ± 12 years and 32 (53.8%) were males. The mean duration of diabetes was 13 ± 6 years, and the HbA1c was 7.5 ± 0.7 gm/dL. Based on fundus examination and FFA, we had 13 patients (21.7%) with normal fundus (Group A), 12 (20%) had NPDR (Group B) and 35 (58.3%) had PDR (Group C). Patients in Group C were significantly older than patients of the other 2 groups. Group C patients also had significantly longer disease duration higher HbA1c level (Table 1). Figure 4 shows the HbA1c level distribution between the 3 groups.

**Table 1:** Comparison of demographic data between Group A, Group B and Group C.

		Fundus exam						*p* value
		Group A		Group B		Group C		
		N	SD/%	N	SD/%	N	SD/%	
Age		42	± 6	53	± 9	65	± 14	** < 0.0001**^**1**^
Duration of diabetes		9	± 2	11	± 5	15	± 7	** < 0.0001**^**1**^
HbA1c		6.6	± 0.4	7.3	± 0.5	7.9	± 0.6	**0.016**^**1**^
Sex	Male	7	53.8%	4	33.3%	21	60.0%	0.27^2^
	Female	6	46.2%	8	66.7%	14	40.0%	

^1^Mann Whitney U test, ^2^Chi2 test.

Significant values are in bold.

### Comparing CDI parameters between Groups A, B and C

OA EDV was significantly higher in Group A with *p* value 0.027, OA RI was significantly higher in Groups B and C compared to Group A with *p* value 0.025. CRA PSV as well as CRA EDV were significantly lower among Group C patients with *p* value 0.017 and 0.001 respectively. PCA RI was significantly higher among Group C patients when compared to Groups A and B, with *p* value 0.008 (Table 2). A post hoc pairwise comparison table was done and also showed significant differences between CDI parameters in different arteries upon comparing each 2 of the 3 groups together (Table 3).

**Table 2:** Comparison of orbital CDI parameters between the 3 groups.

	Fundus exam						*p* value
	Group A		Group B		Group C		
	Mean	SD	Mean	SD	Mean	SD	
OA PSV	30.2	12.0	24.7	10.9	26.4	11.2	0.42
OA EDV	8.8	2.8	5.9	3.7	6.4	2.5	0.027*
OA RI	0.68	0.07	0.74	0.09	0.74	0.08	0.025*
CRA PSV	10.0	2.8	10.0	2.7	7.9	2.6	0.017*
CRA EDV	3.3	1.1	2.9	1.3	2.1	0.7	0.001*
CRA RI	0.69	0.08	0.71	0.07	0.72	0.08	0.31
PCA PSV	11.9	3.4	14.7	5.5	12.7	5.0	0.42
PCA EDV	4.5	1.8	4.0	1.7	3.6	2.0	0.08
PCA RI	0.64	0.06	0.71	0.10	0.72	0.06	0.008*

Kruskal Wallis test.

*OA* Ophthalmic Artery; *CRA* Central Retinal Artery; *PCA* Posterior Ciliary Arteries; *PSV* Peak Systolic Velocity, *EDV* End Diastolic Velocity, *RI*: Resistive Index. *Statistically significant.

**Table 3:** Post-hoc pairwise comparison.

Variable	Group B vs C	Group A vs B	Group A vs C
OA EDV	1.0	0.046*	0.053
OA RI	0.031*	0.077	1.0
CRA PSV	0.088	0.053	1.0
CRA EDV	0.10	0.001*	0.64
PCA RI	0.066	0.007*	1.0

*OA* Ophthalmic Artery; *CRA* Central Retinal Artery; *PCA* Posterior Ciliary Arteries; *PSV* Peak Systolic Velocity, *EDV* End Diastolic Velocity, *RI* Resistive Index. *Statistically significant.

### Correlation between CDI parameters and disease variables

**Figure 5:**
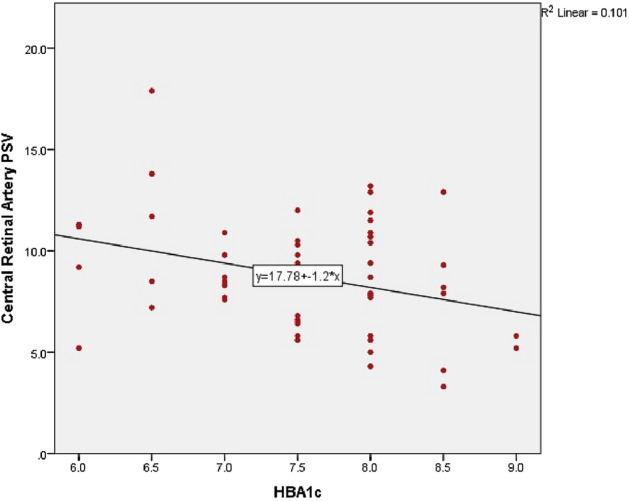
Scatter plot correlating HbA1c level and CRA PSV.

**Figure 6:**
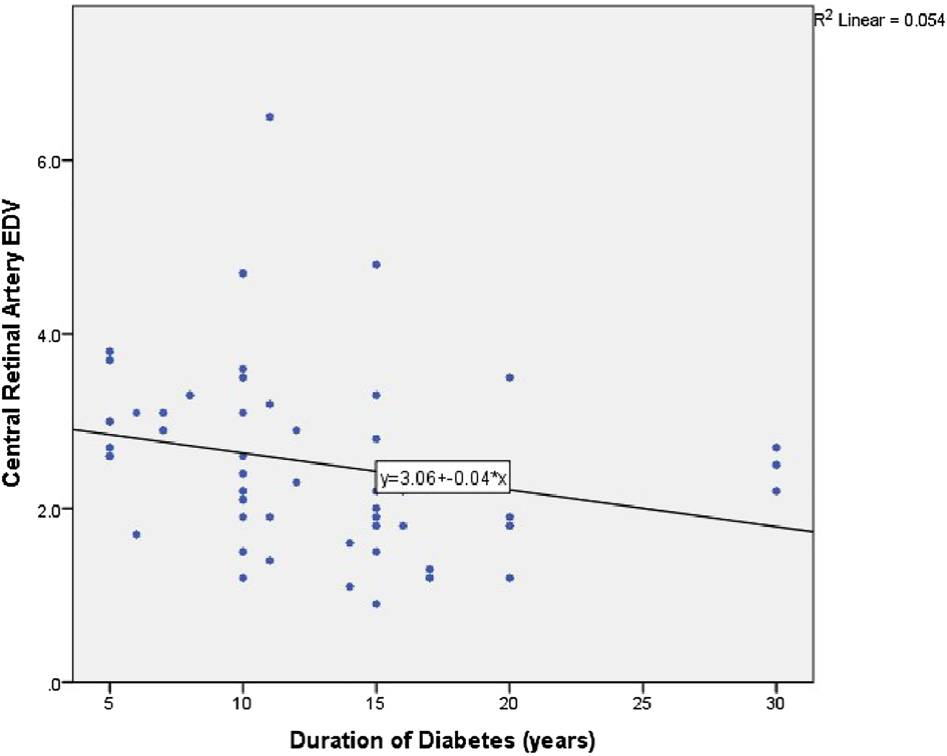
Scatter plot correlating duration of diabetes and CRA EDV.

Interesting correlations between duration of diabetes, HbA1c and CDI findings were found and are shown in Table 4, Figs. 5, 6. HbA1c was negatively correlated with CRA PSV (*p*: 0.041), with CRA EDV (*p*: 0.0001), as well as with PCA EDV (*p*: 0.002). On the other hand, there was direct significant correlation between HbA1c and PCA RI (*p*: 0.012). Disease duration was negatively correlated with CRA EDV (*p*: 0.004).

**Table 4:** Correlation between duration of diabetes, HbA1c and orbital CDI findings.

		Duration of Diabetes	HbA1c
OA PSV	Correlation Coefficient	− 0.104	0.120
	Sig. (2-tailed)	0.429	0.361
OA EDV	Correlation Coefficient	0.077	− 0.003*
	Sig. (2-tailed)	0.560	0.984
OA RI	Correlation Coefficient	− 0.191	0.208
	Sig. (2-tailed)	0.145	0.111
CRA PSV	Correlation Coefficient	− 0.170	− 0.267*
	Sig. (2-tailed)	0.198	0.041*
CRA EDV	Correlation Coefficient	− 0.373**	− 0.468
	Sig. (2-tailed)	0.004*	0.0001*
CRA RI	Correlation Coefficient	0.138	0.184
	Sig. (2-tailed)	0.293	0.160
PCA PSV	Correlation Coefficient	− 0.073	− 0.123
	Sig. (2-tailed)	0.579	0.348
PCA EDV	Correlation Coefficient	− 0.205	− 0.399
	Sig. (2-tailed)	0.116	0.002*
PCA RI	Correlation Coefficient	0.237	0.321
	Sig. (2-tailed)	0.068	0.012*

*OA* Ophthalmic Artery; *CRA* Central Retinal Artery; *PCA* Posterior Ciliary Arteries; *PSV* Peak Systolic Velocity, *EDV* End Diastolic Velocity, *RI* Resistive Index. *Statistically significant.

### Multivariate linear regression models

Multivariate linear regression model was performed with different dependent variables (OA EDV, OA RI, CRA PSV, CRA EDV and PCA RI) and independent variables such as age, sex, duration of diabetes and DR. Results showed that DR is an independent predictor for low OA EDV, high OA RI, low CRA EDV and high PCA RI with *p* values < 0.05 after adjusting for age, gender and duration of diabetes.

## Discussion

### Demographics

In our study, it was noted that patients with higher degree of DR (PDR, Group C) had significantly older age, higher HbA1c, and longer DM duration than the other 2 groups. This is expected with the disease timely progression and in agreement with other studies that reported that aging and higher HbA1c were risk factors for developing DR, and that longer disease duration was consistently found among patients with PDR^9–12^.

### CDI parameters

Orbital CDI indices analysis showed that the OA EDV was significantly lower and OA RI was significantly higher among Group C compared to the other 2 groups. These results are consistent with those reported by Divya et al., who studied 100 diabetic patients, 50 without DR and 50 with DR. They concluded that severe DR was significantly associated with higher OA RI compared to mild DR and diabetics without DR^1^.

We also found that CRA PSV and EDV in Group A patients were significantly higher than Group B and C. This agrees well with what has been reported by Dimitrova et al. and MacKinnon et al., who reported that decreased EDV and increased RI in CRA were consistent with background DR compared to controls^3,13^. But in our case, despite the significant decrease in the CRA flow velocities in DR groups, the increase in RI was not statistically significant. This has been previously concluded in other studies and could be explained by the simultaneous and proportionate decrease of both PSV and EDV in patients with DR^1^.

We also found PCA EDV to be significantly higher in group A, while the PCA RI was significantly highest among Group C compared to Group B, which in its turn was siginifcantly higher compared to Group A. These results agree partially with the results of Divya and his team who stated that PCA EDV and PSV were significantly different between diabetics with and without DR, but their results did not witness any significance regarding RI^1^. The findings of decreased EDV in PCA has been previously explained as a possible result of choroidal blood flow affection in the early stages of DR. An outstanding study by Laser Doppler flowmetry has shown a foveal reduction in choroidal blood flow in early DR^14^. Another study analyzing the Choroidal vascularity index (CVI) using by optical coherence tomography (OCT) has also concluded a decreased CVI in patients with mild/moderate NPDR , which proofs the early choroidal vascular affection^15^. Last but not least, histopathological studies have reported a much more chorio-capillaries loss in diabetic eyes compared to normal eyes^1^.

Since It has been postulated that only 10% or less of the total ocular blood supply from OA flows to the retina while the majority remainder is directed to the ciliary circulation to supply the choroid due to the high metabolic need of photoreceptors^16^, thus our result of increased RI in the OA in PDR group can be attributed to the statistically significant reduction in EDV observed in diabetic subjects caused by distal downstream vascular changes in the choroid mainly and maybe the retina, since significant differences were found in only one CRA parameter between groups. Thus, we believe that the OA hemodynamic alterations in our study mainly represent alteration in choroidal flow, with a minimal affection of retinal blood flow. Other studies found that choroidal flow affection is the main factor in hemodynamic changes and did not find any significant differences in CRA parameters^1^.

Although it is not easy to propose a contributing link, it can be assumed that changes in choroidal circulation (as reflected by high OA RI) lead to upregulation of VEGF (vascular endothelial growth factor) and downregulation of antiangiogenic PEDF (pigment epithelial-derived factor) and hence contributing to DR progression. Variable secretion and expression of these growth factors derived from the retinal pigment epithelium can lead to non-proliferative and proliferative DR^17^.

### Correlations and multivariate analysis

HbA1c was found to be negatively correlated with CRA PSV, CRA EDV, and with PCA EDV. HbA1c also showed significant correlation with PCA RI. These results confirm the direct relationship between poor diabetic control and the hemodynamic alterations in CRA and PCA; and the latter hemodynamic changes were mentioned above to be significantly correlated with the development and severity of DR. This agrees with a previous correlating study stating that duration of diabetes as well as HbA1c levels, were found to be correlating negatively with EDV of CRA^1^. So poorly controlled diabetic patients are more likely to develop DR and to develop hemodynamically significant alteration in orbital arteries. It has been noted in the multivariate linear regression model that we conducted, that DR is an independent predictor for low OA EDV, high OA RI, low CRA EDV and high PCA RI with *p* values < 0.05 after adjusting for age, gender and duration of diabetes.

### Limitations

Our study had limitations, first the relatively small sample which was a reflection of the decreased flow of patients in the Covid-19 period during which this study was conducted. In addition, in the multivariate regression analysis, we used DR as one of the independent variables, but we did not assess the degree of DR as another more detailed variable. Nevertheless, DR was proved to be an independent predictor for some of the CDI parameteric changes in orbital vessels.

In conclusion, DR is an independent predictor for low OA EDV, high OA RI, low CRA EDV, and high PCA RI. CDI can be a useful tool for detecting retrobulbar vascular affection caused by DR. Since alterations in resistivity indices and arterial velocities of the orbital arteries were significantly correlated to DR and HbA1c levels, it thus has the potential to be used as a rapid affordable prognostic screenig tool for the development or the progression of DR by noticing individual changes in flow parameters. We do recommend conducting a larger prospective studies including patients with different grades of DR and to follow them up to assess changes over time, in order to validate and to standardize a protocol to predict the onset or the early progression of DR using CDI.

## Data Availability

All data generated or analyzed during this study are included in this article. Further enquiries can be directed to the corresponding author.
